# The effect of swimming on oral health status: competitive versus non-competitive athletes

**DOI:** 10.1590/1678-775720150324

**Published:** 2016

**Authors:** Simonetta D’ERCOLE, Marco TIERI, Diego MARTINELLI, Domenico TRIPODI

**Affiliations:** Department of Medical, Oral, and Biotechnological Sciences, University “G. D’Annunzio” of Chieti-Pescara, Chieti, Italy

**Keywords:** Cariogenic microorganism, S-IgA, Oral disease, Saliva, Swimming, Sport dentistry

## Abstract

Young swimmers are particularly susceptible to the onset of oral diseases.

**Objective:**

To evaluate the oral health status in young competitive and non-competitive swimmers, involving an assessment of salivary cariogenic bacteria and secretory IgA (S-IgA) concentration.

**Material and Methods:**

Before training sessions (T1), 54 competitive and 69 non-competitive swimmers had the following parameters assessed: decayed, missing, and filled teeth (DMFT), Plaque Index (PlI), and Gingival Index (GI). At T1 and after training sessions (T2), stimulated saliva was collected and microbiological and immunological analyses were performed.

**Results:**

Competitive swimmers trained 2.02±0.09 hours 5 times a week, while non-competitive swimmers trained 2.03±0.18 hours a week. A total of 14.7% of competitive swimmers suffered dental trauma related to sports. Only 11.76% of the competitive swimmers took a daily dose of fluoride, against 32.65% of non-competitive swimmers (p=0.029). Neither group followed an established diet or presented statistically significant differences in terms of nutritional supplement drink and chocolate intake. There were statistically significant differences in terms of oral hygiene. No significant difference in clinical indexes (DMFT, PlI, and GI) was present. *S. mutans* was harbored by 18.6% of competitive and the 32.2% of non-competitive swimmers. *S. sobrinus* was detected in 22.03% of competitive and 91.6% of non-competitive swimmers (p<0.05). *S. sanguinis* was found only in the saliva of competitive swimmers. The average S-IgA of competitive swimmers decreased significantly at T2 (p<0.05). The pool water had a daily average pH of 7.22.

**Conclusions:**

Microbial markers, immune status and sporting characteristics are important for establishing guidelines for management of training load in order to minimize physical stress and the risk of oral infection.

## INTRODUCTION

High performance standards are required for athletes, especially for swimmers, who must be totally healthy individuals. Dental diseases harm quality of life and have a negative impact on self-esteem, eating ability, and health, causing pain, anxiety, and impaired social functioning^4^ ^,^ ^11^ ^,^ ^18^. Relatively little information is available on the correlation between the performance of swimming at a competitive level and tooth decay occurrence^14^. Dental caries is a lifetime disease that depends on biological factors present within the saliva and dental plaque. Dental plaque favors the emergence of *mutans streptococci* and *Lactobacillus* spp., which are capable of rapidly fermenting dietary carbohydrates and lowering the pH to the extent that significant amounts of tooth demineralization can occur^12^ ^,^ ^22^ ^,^ ^23^. The concentration of cariogenic bacteria levels within saliva and plaque determine if caries will occur^24^.

Many different agents protect the tooth surface against developing caries. Secretory IgA (S-IgA) constitutes the main immunoglobulin isotype found in saliva and is considered the first line of host defense against pathogens which colonize or invade the oral cavity. As part of innate immunity, S-IgA is considered the best indicator of mucosal immunity and acts by preventing microbial pathogens from entering the body via mucosal surfaces, which are mainly responsible for infections of the upper respiratory tract^27^ ^,^ ^28^. Several studies have demonstrated that practicing different sports, in particular swimming, at a competitive level, leads to a decrease in S-IgA levels associated with an increased incidence of upper respiratory tract infections in professional athletes^9^ ^,^ ^21^ ^,^ ^24^ ^,^ ^28^ ^,^ ^29^.

The purpose of the present study was to evaluate the oral health of hard and soft tissues and the prevalence of caries in young selected competitive and non-competitive swimmers, by assessing salivary cariogenic bacteria and concentration of salivary IgA, both before and after training sessions. An additional objective was to monitor the water pH of the swimming pool during the day to assess its potential role in erosion.

## MATERIAL AND METHODS

### Study population

The study population was composed of 123 subjects: 54 competitive swimmers (test group) and 69 non-competitive swimmers (control group), 58 females and 65 males. There was no difference between test and control group with respect to age (Table 1). The swimmers (test and control) attended the swimming pools of Chieti and Francavilla al Mare (CH) (Italy) with different training amounts of time (competitive: 2.02±0.09 hours 5 days a week; non-competitive: 2.03±0.18 hours one day a week). All athletes started swimming four years before (4.17±0.27). We discovered that the competitive swimmers trained for the same amount of hours in the swimming pools because the Italian competition rules, edited by the Swimming Italian Federation (FIN), divide the athletes by age and the ones in the same category have an identical training program. This is confirmed by the fact that all athletes belong to the same Society and thus they have the same trainers. The non-competitive swimmers were voluntarily enrolled in the study but had to meet some parameters (training for four years, not belonging to a Sport Society, and not having a planned training program). Parental informed consent was obtained for all patients before they were examined (Privacy Law DL 196/2003). The selected subjects participated voluntarily in the study. In this study, the approval from the Ethics Committee was not required since the research protocol was based on a clinical protocol previously approved by the Department for medical use.

**Table 1:** Demographic and training characteristics of the studied population

	Total number	Male	Female	Mean age	Training table
Competitive swimmers	54	28	26	12.5±3.29	2.02±0.09 hours x 5 times/week
Non-competitive swimmers	69	37	32	9.85±3	2.03±0.18 hours/week

The following inclusion criteria were considered:

Absence of systemic diseases that could affect the immune response or that could condition bacterial colonization.

No use of antibiotics for three months prior to the beginning of the study.

No orthodontic therapy.

Dental care not in progress.

A self-administered questionnaire was used to obtain data concerning hours and frequency of weekly training, complete pathological history, history of hard and soft tissues of the oral cavity, family history, oral hygiene practices, fluoride intake, and eating habits (supplements consumed and dietary information such as intake of drinks, fruit juices, and consumption of chocolates).

### Clinical monitoring and saliva collection

Before training sessions (T1), each patient was clinically evaluated. The number of decayed, missing, and filled teeth (DMFT) was recorded to assess the prevalence of caries according to World Health Organization (WHO) criteria. The Silness *&* Löe Plaque Index (PlI) and the Löe & Silness Gingival Index (GI) were used to evaluate oral hygiene and periodontal status, respectively.

In addition, an oral examination of intraoral mucosa was performed and the presence/absence of bad habits, malocclusion, and parafunctional habits was assessed.

Before (T1) and after training sessions (T2), saliva was collected with paraffin-chewing stimulation. The saliva samples were immediately placed on ice and transferred to the laboratories for processing. An aliquot of 500 μl from each sample was transferred to a microcentrifuge tube and used for microbiological analysis. The remainder of the sample was placed in an eppendorf tube, clarified by centrifugation at 10,000 Xg for 15 min at 4°C and frozen at -20°C for the titration of salivary IgA.

### DNA extraction from saliva samples

The presence of three oral streptococcal species (*Streptococcus mutans*, *Streptococcus sobrinus*, and *Streptococcus sanguinis*) in each saliva specimen was analysed by PCR. *S. mutans* ATCC31383, *S. sobrinus* ATCC27607, and *S. sanguinis* 49297 were used as control species. The sequences of species-specific sets of the primers used in this study are listed in Table 2. Saliva samples were stored at -20°C until DNA extraction (24-48 h later). Vortexed saliva samples were centrifuged for 15 min at 14000 rpm. Next, the supernatants were discarded and individual cell pellets were stored at -20°C until DNA isolation, for which the pellets were resuspended in 300 μl lithic solution (50 mM Tris, 10 mM EDTA, 10% SDS), treated with lysozyme (5 mg/ml) and incubated for 1 h at 37°C. Proteinase K was added and incubated for 1 h at 65°C, DNA was extracted according to the phenol:chloroform:isoamyl alcohol method. Nucleic acids were precipitated in alcohol, washed with 70% (vol/vol) alcohol, and suspended in double-distilled water to create the DNA template.

**Table 2:** Polymerase chain reaction primers

Bacterial species	Primer pairs 5’-3’	Amplification length	Reference
*S. mutans*	5’- GGC ACC ACA ACA TTG GGA AGC TCA GTT	433 bp	17
	5’- GGA ATG GCC GCT AAG TCA ACA GGA T		
*S. sobrinus*	5’- GAT GAT TTG GCT CAG GAT CAA TCC TC	328 bp	17
	5’-ACT GAG CCA GTA GTA GAC TTG GCA ACT		
*S. sanguinis*	5’- GGA TAG TGG CTC AGG GCA GCC AGT T	313 bp	17
	5’-GAA CAG TTG CTG GAC TTG CTT GTC		
Ubiquitous primer	5’-GAT TAG ATA CCC TGG TAG TCC AC	602 bp	7
	5’-CCC GGG AAC GTA TTC ACC G		

### Polymerase chain reaction

Negative controls (no DNA) and positive controls with DNA coming from pure bacterial cultures were tested. Amplification was performed in 100 μl reaction, containing 10 mM Tris-HCl, pH 8.0, 50 mM KCl (1 x PCR Buffer), 1.5 mM MgCl, 200 μM of each nucleotide, 30 ml of each primer, 2.5 U Hot Start Taq DNA Polymerase (Quiagen S.P.A. Milan Italy) and 5 μl of DNA Template. The reaction mixture was denatured at 95°C for 3 min, followed by 30 cycles of denaturation at 98°C for 30 sec, annealing at 70°C for 30 sec, and extension at 72°C for 1 min. The reactions were conducted in a thermocycler iCyclar System (Bio-Rad Laboratories srl, Segrate, Italy). Amplification of ubiquitous primers was conducted in the same way. Electrophoretic bands were visualized and photographed with a UV rays transilluminator (gel Doc 2000, Bio-Rad) after staining for 30’ with ethidium bromide (1 μg/ml). The sizes of the PCR products were estimated from the electrophoretic migration of products relative to a 100-base-ladder marker (Amersham Pharmacia Biotech, AB, Uppsala, Sweden).

### Quantification of total IgA

The titration of salivary IgA was performed by using LC-examination Partigen immunodiffusion (Dade Behring Marburg GmbH, Germany).

### Swimming pool water collection

Samples of swimming pool water (10 ml) were also collected in test tubes at regular intervals throughout the day and sent to the laboratory for pH evaluation. pH values were analyzed by a pHmeter (Elettrofor, Scientific Instruments, Borsea, Italy).

### Statistical analysis

The Wilkoxon matched-pairs signed-rank test was used for the statistical analysis. All p-values lower than 0.05 were considered significant.

## RESULTS

The demographic and clinical characteristics of the studied populations are presented in Table 1 and Table 3. The competitive swimmers trained 2.02±0.09 hours 5 times a week and the non-competitive swimmers 2.03±0.18 hours a week. Age and gender were similar in the two groups. While 14.7% of competitive swimmers had suffered dental trauma related to sports, there were no cases in the control group. Only 11.76% of the competitive swimmers took a daily dose of fluoride against 32.65% of non-competitive swimmers, with a statistically significant difference.

**Table 3:** Prevalence (%) of clinical characteristics and eating habits between genders

	Competitive swimmers	Non-competitive swimmers
Dental stains	11.76	2.04
Dental erosion	2	1
Dental trauma	14.7	0
Oral habits	2.94^§^	28.57^§^
P = 0.003 S		
Aphthae	8,8	16.3
Decay presence	38.24	42.86
Fluoride intake	11.76^§^	32.65^§^
P = 0.029 S		
Chocolate intake	20.59^§^	59.18^§^
P = 0.000 S		
Sport drinks intake	84.62^§^	15.38^§^
P = 0.000 S		
Fruit intake	17.65	8.16
Dental anomalies	44.12	59.18

Statistical significance of the differences between the groups: §, p<0.05

Data concerning the dietary habits showed that neither group followed an established diet and that their nutritional supplement drink and chocolate intakes were significantly different (p=0.000).

Statistically significant differences in oral habits between the two groups were observed. The most frequently reported by the study population were nail biting, sleep bruxism, and atypical swallowing.

The analyzed clinical indexes (DMFT, PlI, GI) did not present statistically significant differences (Table 4). It is noteworthy that 94.12% of competitive swimmers presented values of PlI lower than 1.5 against 77.55% of the non-competitive swimmers, a statistically significant difference (Table 5).

**Table 4:** Clinical parameters of the experimental groups at baseline

	Competitive swimmers	Non-competitive swimmers
DMFT	0.0822187±0.1014739	0.10857±0.12798
Plaque Index (PlI)	0.607±0.539	0.7476±0.7189
Gingival Index (GI)	0.0493±0.186	0.0611±0.121

Statistical significance of the differences between the groups: *, p<0.05.

**Table 5:** Clinical indexes in the studied population

	Competitive swimmers	Non-competitive swimmers
Plaque Index (PlI)	94.12%*	77.55%*
Gingival Index (GI)	8.82%	20.41%

Statistical significance of the differences between the groups: *, p<0.05.

As shown in Table 6, 18.64% of competitive and 32.2% of non-competitive swimmers harbored *S. mutans* in their saliva. *S. sobrinus* was present in 22.03% of competitive swimmers and 91.6% of non-competitive swimmers, with a statistically significant difference between the two groups. On the other hand, *S.sanguinis* was determined only in the saliva sample of the competitive swimmers. No difference was noted between pre- and post-training samples.

**Table 6:** Percentage (%) of saliva samples from the studied population with presence of the main microorganisms examined (pre- and post-training)

	Competitive swimmers	Non-competitive swimmers
*S. mutans*	18.64	32.2
*S. sobrinus*	22.03	91.6 ^†^
*S. sanguinis*	10.16	0

Statistical significance of the differences between the groups or time examinations: †, p<0.05

Competitive swimmers showed a statistically significant decrease of the average value in the titration of S-IgA (Table 7). The average values of the competitive swimmers were significantly higher than of the non-competitive swimmers.

**Table 7:** Salivary IgA (S-IgA) concentrations in saliva samples collected from competitive and non-competitive swimmers

	S-IgA Pre-training	S-IgA Post- training
Competitive swimmers	0.091087±0.2218976	0.0443333±0.0167551*
Non-competitive swimmers	0.035087±0.0049992**	0.0347826±0.0044206**

Statistical significance of the differences between the groups or time examinations: *,**, P < 0.05

* vs pre-training

** vs competitive

The waters samples from the pools had a daily average pH value of 7.22 and both swimming pools followed hygiene recommendations, which included a double daily pH register, daily assessment of antimicrobial concentration in the water, quantity of purified and renewed water, and water and environmental temperatures.

## DISCUSSION

The exercise involved in routine sport practice exposes athletes to the onset of numerous diseases. A number of factors, including the levels of competition and of exposure, can affect the higher or lower incidence. For this reason the present study considered two groups of swimmers attending the same environment (same pools) and same years of practice (4.17±0.27), but with different training loads. Results from this study indicated that athletes engaged in swimming at a competitive level, with more hours of training in pool water, had a higher incidence of dental stains and trauma. Dental injuries associated with various types of sports have often been described in literature^6^ ^,^ ^10^ ^,^ ^19^ ^,^ ^30^.

The incidence of dental stains was higher in competitive swimmers compared to non-competitive ones. Although the difference was not statistically significant, it can be caused, as claimed by Escartin, et al.^11^ (2000), by the chemical used to disinfect the pool water in both swimming pools analyzed and by the time spent inside swimming pools and performing sufficiently vigorous physical exercise in order to permit contact between water and dental surfaces. In this study, the amount of training time of the competitive swimmers (2.02±0.09 hours 5 times a week) falls under the condition mentioned by Escartin, et al^11^(2000) of more than 6 hours of training per week as the time necessary to increase the risk of dental stains. Although the values are much lower than those reported by Escartinet, al^11^(2000) (prevalence of Dental Stains (DS) of 60.2% in the swimmers), it is possible to state that competitive swimmers had a high risk of developing extrinsic dental stains.

Competitive swimmers had a low prevalence of parafunctional behaviors. The most frequent oral habits reported by non-competitive swimmers were nail biting, sleep bruxism, and atypical swallowing, with an incidence similar to those reported in young adolescents in the literature^15^.

Data concerning the dietary habits showed that in both groups, because of the age of the sample, no one followed a specific diet for training or competitions. Intake of nutritional supplement drinks by competitive swimmers was significantly different from non-competitive swimmers. Dental erosion is a further potential problem associated with sports drinks^1^ ^,^ ^5^ ^,^ ^14^. This study showed no potential problems related to the consumption of these beverages, as demonstrated by low dental erosion frequency.

Some authors believe that if the pH of the water in the pool decreases below that of saliva, erosions on the dental enamel may occur^3^ ^,^ ^18^. In this study, the waters of the pools showed a daily pH of 7.2, thereby meeting the required pH values ranging from 7.20 to 9.0 and having little or no effect on swimmers in relation to the development of erosions (2% *vs* 1%).

In this study, after training, there was a statistically significant decrease in the concentration of salivary IgA levels of selected swimmers. S-IgA average values before and after training were significantly higher in competitive athletes than in non-competitive swimmers. The results of this study confirmed the majority of studies in that acute bouts of exercise reduced S-IgA levels in a variety of endurance sports, including swimming^7^ ^,^ ^8^ ^,^ ^16^ ^,^ ^25^ ^,^ ^28^. These results contrasted with some authors who indicated that there are no differences in salivary concentrations of S-IgA between athletes and non-athletes, except when athletes are engaged in heavy training^13^ ^,^ ^25^. Several studies demonstrated that S-IgA concentration changes and in particular an absolute concentration of less than 40 mg/L was reported to relate to increased Upper Respiratory Tract Infection (URTI) incidence in athletes^28^. Therefore, since S-IgA acts as a marker of mucosal immunity, this study showed that its decline could be associated with a decrease in host defenses, and specifically of the mouth, with possible negative consequences on health status.

The microbiological analysis showed that only 18.36% and 22.03% of competitive swimmers harbored *S. mutans* and S*. sobrinus* in their saliva*,* respectively. On the other hand, these bacteria were detected in 32.2% and 91.6% of non-competitive swimmers. Moreover, *S. sanguinis* was determined only in the saliva sample of the competitive swimmers. Mutans streptococci (*S. mutans* and *S. sobrinus*) are closely associated with the development of dental caries in humans. *S*. *sanguinis* was significantly associated with dental health, and the results of this study confirmed that *S. sanguinis* has been reported to be in relatively high numbers when the numbers of *S. mutans* are low, as reported in competitive swimmers. Moreover, in competitive swimmers the presence of high numbers of *S. sanguinis* was associated with low caries risk, as suggested by Seow, et al.^26^ (2009). A high percentage of non-competitive swimmers harbored cariogenic bacteria and their growth is favored by eating habits such as a large intake of chocolate.

Therefore, the results suggested that the time of training was the period characterized by the least intensive salivary functions and physiologic responses as a decrease in the salivary level of IgA. Swimming at competitive levels determined higher S-IgA average values, both before and after training, than for non-competitive swimmers. A low number of competitive swimmers harbored cariogenic microorganisms in their saliva; on the contrary, they showed a higher frequency of “protective” bacteria as *S. sanguinis*, associated with low caries risk, as confirmed by lower values of active caries. Several studies^2^ ^,^ ^20^ reported that the oral health of those who practice the sport at a competitive level was harmed, with a high incidence of oral problems such as caries, trauma, etc. The difference from the results of this study was the age of the examined subjects, proving it is crucial to avoid the problems described in the literature by implementing a prevention program.

## CONCLUSIONS

This study confirms the usefulness of using microbial risk markers, including mutans streptococci. Non-microbial caries risk factors included visible plaque, child age, race, salivary and pool water pH, and eating habits. Furthermore, mucosal immune status and sport characteristics (intensity, duration and frequency of training) are considered useful for establishing guidelines for nutritional support and management of training load. In this way, it would be possible to minimize physical stress and subsequently the risk for oral infection.
